# P62‐positive aggregates are homogenously distributed in the myocardium and associated with the type of mutation in genetic cardiomyopathy

**DOI:** 10.1111/jcmm.16388

**Published:** 2021-02-18

**Authors:** Zoë Joy van der Klooster, Shahrzad Sepehrkhouy, Dennis Dooijes, Wouter P. te Rijdt, Frederique S. A. M. Schuiringa, Jolanthe Lingeman, Johannes Peter van Tintelen, Magdalena Harakalova, Roel Goldschmeding, Albert J. H. Suurmeijer, Folkert W. Asselbergs, Aryan Vink

**Affiliations:** ^1^ Department of Pathology University Medical Center Utrecht Utrecht University Utrecht The Netherlands; ^2^ Department of Genetics University Medical Center Utrecht Utrecht University Utrecht The Netherlands; ^3^ Department of Genetics University Medical Center Groningen University of Groningen Groningen The Netherlands; ^4^ Department of Cardiology University Medical Center Utrecht Utrecht University Utrecht The Netherlands; ^5^ Department of Pathology University Medical Center Groningen University of Groningen Groningen The Netherlands; ^6^ Health Data Research UK and Institute of Health Informatics University College London London UK; ^7^ Institute of Cardiovascular Science Faculty of Population Health Sciences University College London London UK

**Keywords:** autophagy, cardiomyopathy, desminopathy, genetic, histology, P62, pathology, phospholamban, senescence, sequestosome‐1

## Abstract

Genetic cardiomyopathy is caused by mutations in various genes. The accumulation of potentially proteotoxic mutant protein aggregates due to insufficient autophagy is a possible mechanism of disease development. The objective of this study was to investigate the distribution in the myocardium of such aggregates in relation to specific pathogenic genetic mutations in cardiomyopathy hearts. Hearts from 32 genetic cardiomyopathy patients, 4 non‐genetic cardiomyopathy patients and 5 controls were studied. Microscopic slices from an entire midventricular heart slice were stained for p62 (sequestosome‐1, marker for aggregated proteins destined for autophagy). The percentage of cardiomyocytes with p62 accumulation was higher in cardiomyopathy hearts (median 3.3%) than in healthy controls (0.3%; *P* < .0001). p62 accumulation was highest in the desmin (15.6%) and phospholamban (7.2%) groups. P62 accumulation was homogeneously distributed in the myocardium. Fibrosis was not associated with p62 accumulation in subgroup analysis of phospholamban hearts. In conclusion, accumulation of p62‐positive protein aggregates is homogeneously distributed in the myocardium independently of fibrosis distribution and associated with desmin and phospholamban cardiomyopathy. Proteotoxic protein accumulation is a diffuse process in the myocardium while a more localized second hit, such as local strain during exercise, might determine whether this leads to regional myocyte decay.

## 1 INTRODUCTION

The intracellular accumulation of misfolded proteins and damaged cell organelles is increasingly reported in heart failure. In the normal situation, protein degradation pathways, like autophagy, prevent this accumulation of misfolded proteins. Recent studies suggest an important role for impaired autophagic processes in myocardial diseases such as myocardial infarction, pressure overload hypertrophy and various forms of cardiomyopathies.^1^

Autophagy regulates cellular homoeostasis by transporting intracellular cargo to the lysosomes for bulk degradation and recycling of macromolecules.^1^ This process reduces proteotoxic stress and delivers new building blocks and energy for the cell. In macroautophagy, intracellular vesicles, also known as autophagosomes, are formed containing damaged macromolecules (eg mutant, misfolded or damaged proteins) and organelles (eg mitophagy). These autophagosomes travel along microtubules and then fuse with lysosomes which are then termed autophagolysosomes. After fusion with lysosomes, the intracellular cargo is degraded by lysosomal enzymes and reused.^2^

P62 recognizes ubiquitinated proteins and promotes aggregation and sequestration (synonym sequestosome‐1) marking protein aggregates for degradation by autophagy or the ubiquitin proteasome system.^3^ After fusion of the autophagosome with the lysosome, p62 is efficiently degraded by lysosomal enzymes. When autophagy is unable to remove the p62 bound proteins, p62 accumulates in the cell. Accumulation of p62‐positive aggregates in the cell may therefore be a measure of the amount of proteotoxic aggregates. These aggregates may in turn activate pathways leading to cardiomyocyte dysfunction, including cellular senescence.^4^

In hearts of cardiomyopathy patients with a pathogenic variant in the gene encoding phospholamban (*PLN*), we have recently demonstrated aggregates that are both p62 and phospholamban positive.^5^ This suggests that the autophagy system is not able to remove all the mutant phospholamban protein, resulting in accumulation of p62‐positive proteotoxic perinuclear aggregates in the cardiomyocytes. p62 accumulation has also been described in some other genetic cardiomyopathies.^6^ In the present study, we investigated the presence of p62 proteotoxic aggregates in genetic cardiomyopathies caused by pathogenic mutations in different cardiomyopathy‐related genes (*DES, CRYAB, PLN, PKP2, DSP, LMNA, MYBPC3, MYH7, TNNI3 and TNNT2*). In addition, we studied the distribution of these aggregates throughout the myocardium.

## 2 METHODS

The study met the criteria of the code of proper use of human tissue that is used in the Netherlands. The collection of the human heart tissue was approved by the scientific advisory board of the biobank of the University Medical Center Utrecht, Utrecht, the Netherlands (protocol no. 12/387). Written informed consent for collection and biobanking of tissue samples was obtained prior to transplantation or, in certain cases, approved by the ethics committee when obtaining informed consent was not possible because of death of the patient.

Hearts from 32 patients with a genetic cardiomyopathy and 4 patients without known pathogenic mutations were included in the study that were obtained during heart transplantation or at autopsy. Genetic testing was performed in a clinical setting. Patients were classified in functional protein groups encoded by (likely) pathogenic variants (mutations) in genes encoding desmin (*DES*) and the desmin‐related αB‐crystallin (*CRYAB*), phospholamban (*PLN*), desmosomal proteins (*PKP2* and *DSP*), lamin A/C (*LMNA*), and sarcomeric proteins (*MYBPC3, MYH7, TNNI3 and TNNT2*). Five control hearts were used (four donor hearts not used for transplantation and one heart acquired at autopsy after a non‐cardiac death). At the time of clinical presentation, the cardiomyopathy subtype of these patients had been classified by their cardiologist as arrhythmogenic [ACM] according to the revised task force criteria,^7^ dilated [DCM] or hypertrophic [HCM] cardiomyopathy.

A complete heart slice halfway between the apex and the atrioventricular valves was formalin‐fixed and divided into smaller pieces as described previously.^8^, ^9^ The formalin‐fixed heart tissue was embedded in paraffin and cut into 4 µm sections. Hematoxylin and eosin (H&E) and Masson's trichrome stains were done to evaluate general morphology and fibrosis.

**FIGURE 1:**
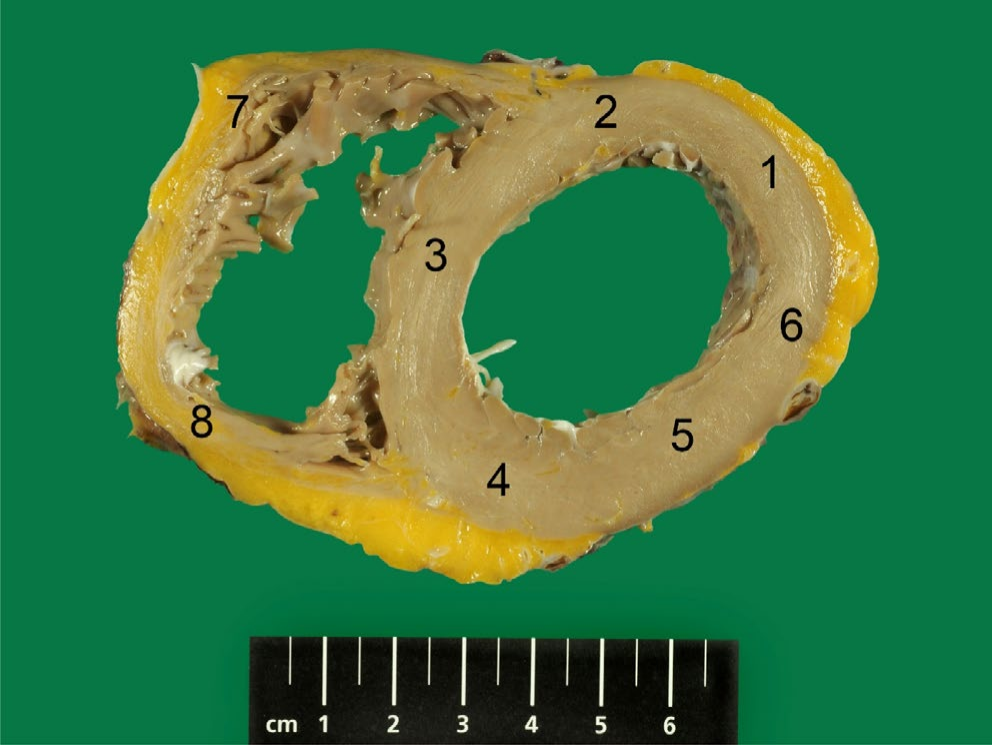
Example of heart slice of PLN cardiomyopathy heart divided into 8 regions: 5 regions for the left ventricular free wall (anterior, anterolateral, lateral, posterolateral, posterior), 1 region for the septum and 2 regions for the right ventricle (posterior and anterior). Bottom of figure is anterior part of the heart

A mouse anti‐p62 Ick ligand antibody (BD Biosciences) was used to stain for p62 using the Ventana BenchMark Ultraplatform (Roche). For p62 quantification, the heart slice was divided into five regions for the free wall of the left ventricle (LV; anterior, anterolateral, lateral, posterolateral and posterior), one region for the septum and two regions for the right ventricle (RV; posterior and anterior) as we have described previously (Figure 1).^8^, ^9^ Every region was then split into three areas: trabeculated, inner compact (middle layer) and outer compact (epicardial) myocardium. In all cases, a hot spot count was done in the different areas: 250 cells were counted per area (8 regions x 3 areas x 250 cells = 6000 counted cells per heart). In the phospholamban group, the amount of fibrosis was also digitally quantified in the 6 regions of the heart in the three areas (trabeculated, inner and outer compact) as described previously.^8^, ^9^

### 2.1 Statistics

Data are expressed as median [interquartile range]. A Mann‐Whitney test was used to compare percentages between two groups and a Kruskal‐Wallis test to compare multiple groups. Dunn's multiple comparison test was used to correct for multiple comparisons. Linear regression was used to test the association between two continues variables.

## 3 RESULTS

### 3.1 Patient characteristics

Patient characteristics are summarized in Table 1. The mean age of cardiomyopathy patients at cardiac transplantation or autopsy was 46 ± 14 years; 22 patients (61%) were male. The initial clinical cardiomyopathy diagnosis registered by the treating cardiologist varied among groups. All patients had severe end stage heart failure. Half of the patients (52%) had undergone implantation of a left ventricle assist device (LVAD) before the heart was obtained at transplantation or autopsy. The genetic variants (mutations) of all patients are provided in the Supplementary Table S1.

**TABLE 1:** Patient characteristics per pathogenic genetic mutation group

	Desminopathy n = 3	Phospholamban n = 9	Desmosomal n = 6	Lamin A/C n = 7	Sarcomeric n = 7	No mutation n = 4
Age (year ± SD)	52 ± 6	46 ± 13	54 ± 8	49 ± 15	31 ± 9	52 ± 15
Sex (m/f)	2/1	4/5	4/2	6/1	4/3	2/2
Initial clinical diagnosis	1 ACM 2 DCM	2 ACM 7 DCM	1 DCM 5 ACM	7 DCM	3 HCM 4 DCM	4 DCM
LVAD, n (%)	2 (67%)	6 (67%)	0 (0%)	5 (71%)	3 (43%)	3 (75%)
Explant / autopsy	2/1	6/3	5/1	6/1	7/0	3/1

Note

Abbreviations: ACM, arrhythmogenic cardiomyopathy; Age, age at transplantation or autopsy; DCM, dilated cardiomyopathy; f, female; HCM, hypertrophic cardiomyopathy; LVAD, left ventricular assist device; m, male; SD, standard deviation.

### 3.2 Differences between mutation groups

**FIGURE 2:**
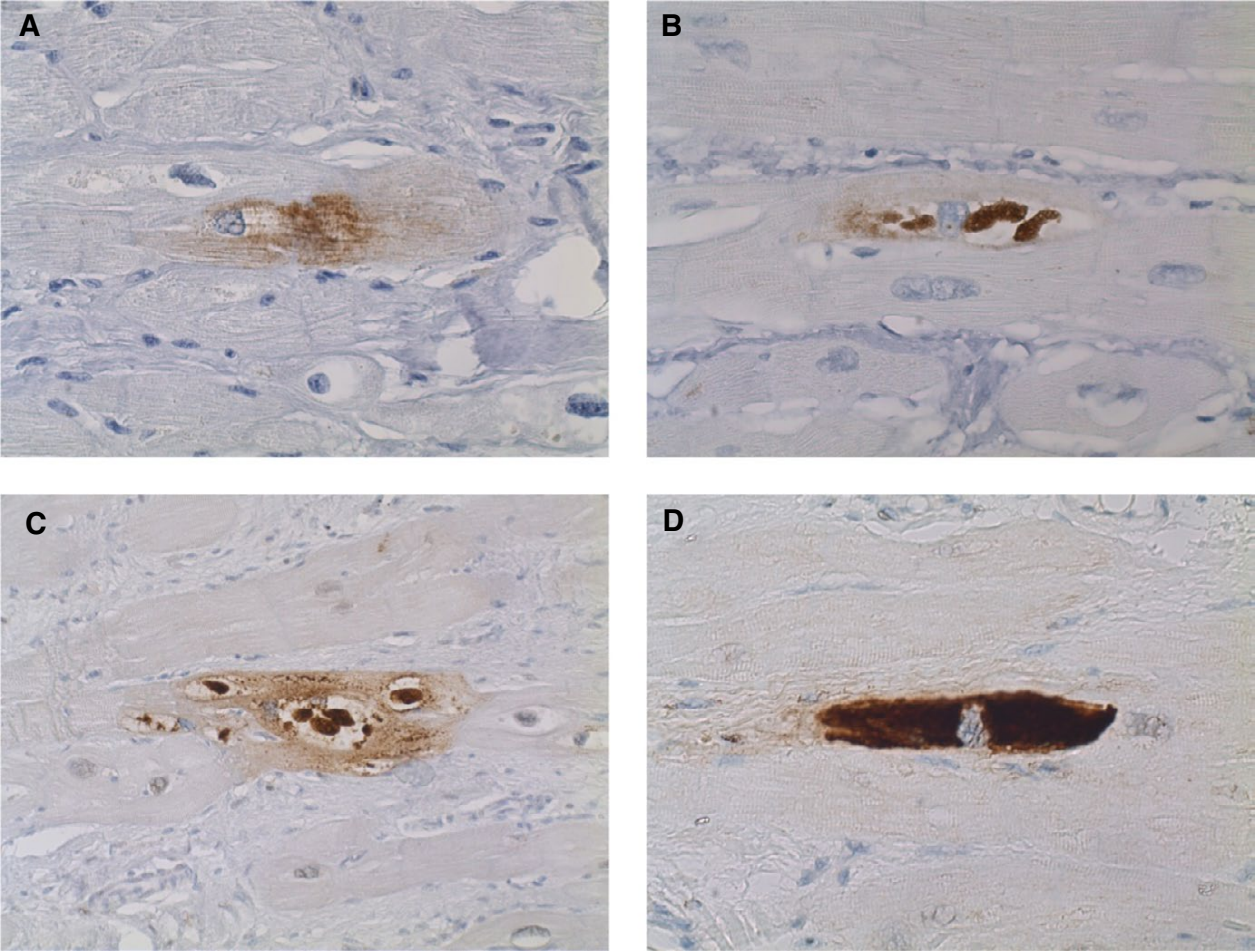
Staining patterns of p62 in cardiomyocytes. A, diffuse staining of the sarcolemma without obvious aggregates. B, perinuclear p62‐positive aggregates. C, p62‐positive aggregates expand into the sarcolemma. D, p62‐positive aggregates occupy almost the complete sarcolemma of the cardiomyocyte. Magnification 400×

**FIGURE 3:**
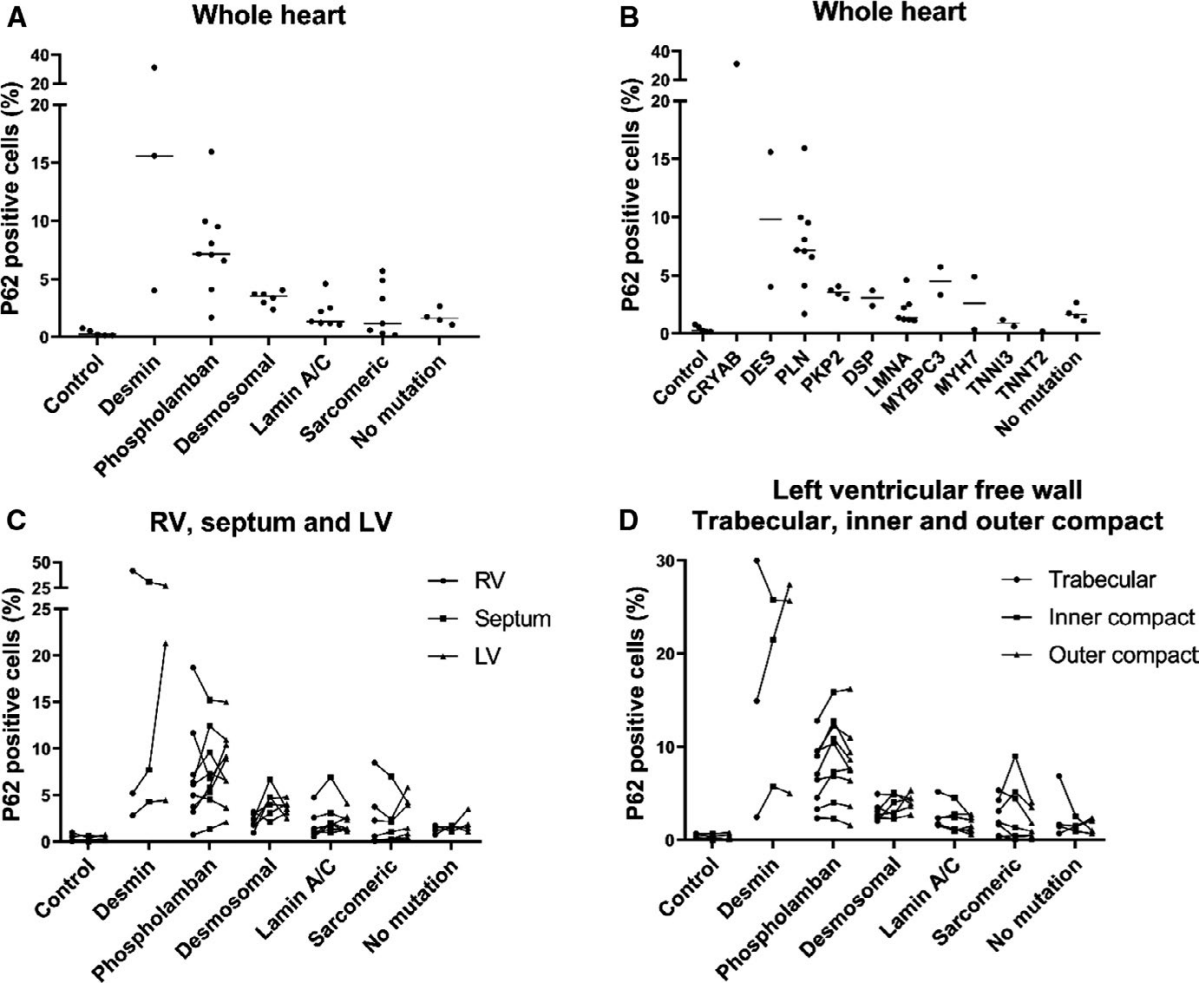
Percentage cardiomyocytes with p62‐positive aggregates per gene and mutation group. A, percentage p62‐positive cardiomyocytes per mutation group in the whole heart. The desmin and phospholamban mutation groups showed significantly higher percentages as compared to the controls and other groups. B, percentage p62‐positive cardiomyocytes per gene in the whole heart. C, percentage p62‐positive cardiomyocytes in right ventricle (RV), septum and left ventricle (LV). In the desmosomal group, the percentage of p62‐positive cardiomyocytes was significantly lower in the RV than in the septum and LV. No difference between RV, septum and LV was observed in the other groups. D, no difference between percentage p62‐positive cardiomyocytes in the trabecular and inner and outer compact layer of the LV was observed

p62 showed a globular staining pattern of small or larger perinuclear aggregates or diffuse staining of the sarcolemma in the majority of cases, which is in concordance with our previous observation (Figure 2).^5^ In some cases, the aggregates were present in large parts of the sarcolemma with very limited remaining sarcomeres. In the healthy control group, a median of 0.3% [0.1‐0.7] of cardiomyocytes revealed p62 accumulation *versus* 3.3% [1.4‐6.4] in the cardiomyopathy hearts (*P* < .0001). In the cardiomyopathy hearts, p62 accumulation was highest in the desmin, phospholamban and desmosomal gene groups (15.6% [4.0‐31.3], 7.2% [5.3‐9.7] and 3.5% [2.8‐3.8], respectively) followed by, lamin AC (1.3% [1.2‐2.5]) and sarcomeric gene (1.2% [0.3‐4.9]) groups (Figure 3A). P62 accumulation in the latter two groups was comparable with hearts without known mutations (1.6% [1.2‐2.4]). Both the desmin and phospholamban hearts showed significantly higher percentages of cardiomyocytes with p62 accumulation as compared to the other groups. Within the desmosomal group, percentages of p62‐positive cells were comparable for the *PKP2* and *DSP* gene mutations (Figure 3B).

### 3.3 Differences between RV, septum and LV

In the whole group of cardiomyopathy hearts, no significant differences were observed between the percentage of p62‐positive cells in the RV (2.3% [0.9‐4.9]), septum (3.1% [1.5‐6.8]) and LV (3.6%[1.4‐6.4]). In the desmosomal gene group, the percentage of p62‐positive cells in RV (2.1% [1.6‐3.1]) was significantly lower than the percentages in the septum (4.3% [2.8‐5.3]) and LV (3.7% [2.9‐4.2]; *P* = .02). In the subgroup analysis of the other mutation groups, no significant difference was observed between RV, septum and LV (Figure 3C).

### 3.4 Difference between trabecular, inner and outer compact myocardium in RV and LV

Both in the cardiomyopathy hearts pooled group and in the different mutation groups, no differences were observed between the trabecular, inner and outer compact myocardium for RV, LV, RV and LV combined and posterolateral wall of the LV (Figure 3D).

### 3.5 P62 is not associated with the amount of fibrosis in phospholamban hearts

Given the relatively high numbers of phospholamban hearts, we decided to perform a subgroup analysis to study the association between p62 and fibrosis. The percentage of p62‐positive cardiomyocytes was not associated with the percentage fibrosis in the myocardium of the whole heart (*P* = .8), RV (*P* = .9), LV (*P* = .9) and the three separate layers of the left ventricular wall (*P* = .7, 0.8 and 0.6 for trabulated, inner and outer compact myocardium, respectively).

## 4 DISCUSSION

The accumulation of potentially proteotoxic protein aggregates due to insufficient autophagy is a possible contributing mechanism of disease development in genetic cardiomyopathy.^1^, ^4^ We studied the myocardial distribution of cardiomyocyte autophagosomes in different genetic cardiomyopathies using p62, a marker for aggregated proteins. This study has yielded two important results: first, the accumulation of potentially proteotoxic aggregates in cardiomyocytes differs with the specific underlying pathogenic genetic mutation. The highest amounts of aggregates were observed in desmin and phospholamban cardiomyopathy hearts. Second, the distribution of these aggregates is homogeneously distributed in the myocardium independently of fibrosis distribution with the only exception of the desmosomal gene variant group where the RV revealed less aggregates.

The highest percentages of cardiomyocytes with aggresomes were observed in desmin cardiomyopathy patients, including one patient with a pathogenic mutation in the desmin‐related αB‐crystallin (*CRYAB*) gene. This observation is in line with the classical histopathological hallmark of many desmin‐related cardiomyopathies, that is abnormal cytoplasmic desmin aggregation in cardiomyocytes.^10^, ^11^ In a cell culture study, homogenous infection of different cell types with the *DES* p. Asn342Asp variant (15% p62‐positive cells in our study) induced cytoplasmic aggregates and small desmin fibrils without normal filament assembly. However, in a heterozygous co‐expression experiment, that is more comparable to the human situation, mutant and wild‐type desmin molecules were incorporated into the same filamentous structures without aggregate formation.^12^ The present study shows that also in the heterozygous human situation during life aggregates develop within cardiomyocytes throughout the myocardium. Our observation is also in concordance with previous observations in a desmin and αB‐crystallin cardiomyopathy mouse model where increased cardiomyocyte p62 was demonstrated. In this model, p62 appeared to promote aggresome formation and autophagy of misfolded proteins in order to protect cardiomyocytes against proteotoxic stress.^13^ There is an ongoing debate regarding whether these desmin aggregates are toxic or the disturbed intermediate filament network is the molecular trigger for the degeneration of the cardiomyocytes.^10^ We observed sarcoplasmic aggregates in up to 30% of cardiomyocytes in *CRYAB* and 15% in *DES* cardiomyopathy replacing large parts of the sarcomeres, suggesting that these aggregates may play an important role in the impaired myocardial function of these cardiomyopathies. In addition, it has been demonstrated that aberrant protein aggregation in desmin cardiomyopathy leads to mitochondrial dysfunction, abnormal metabolism, and altered cardiomyocyte structure.^14^

**FIGURE 4:**
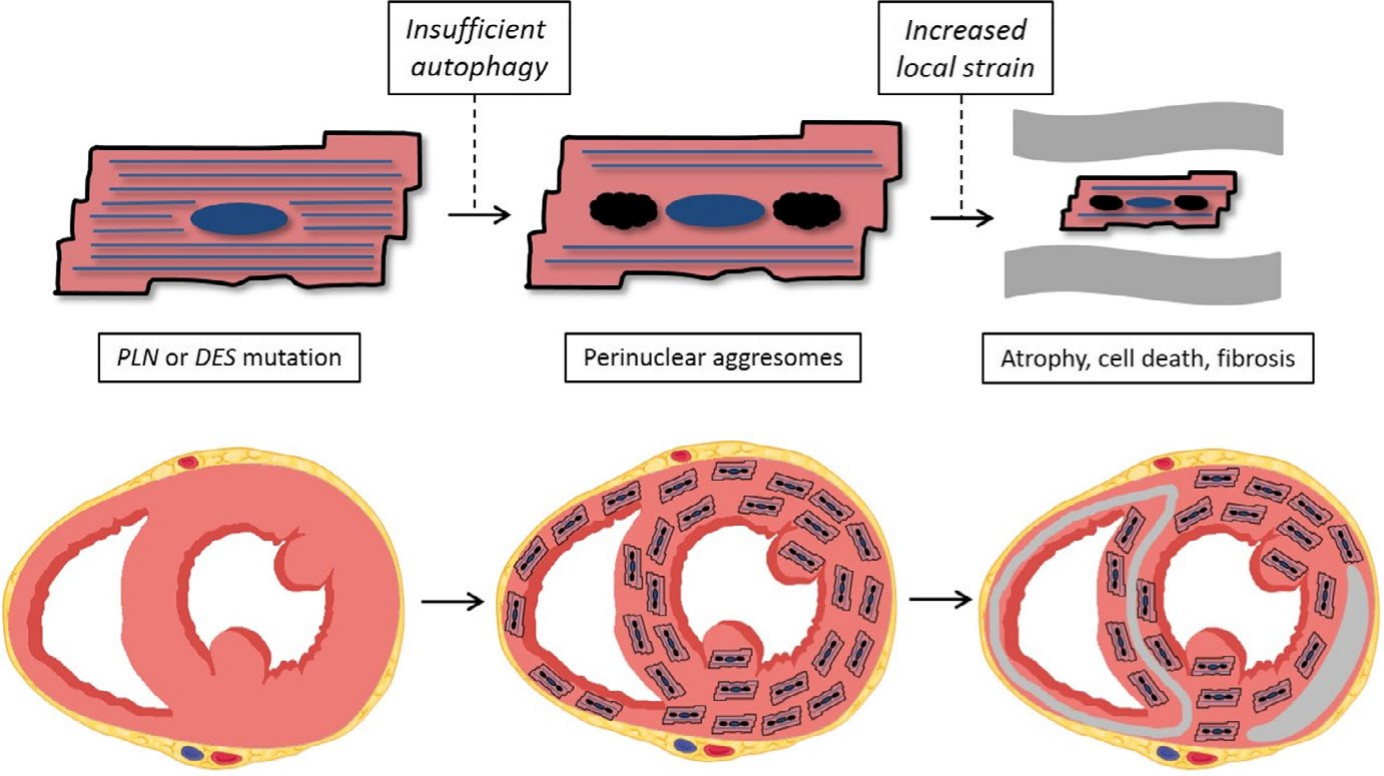
Hypothesis of pathophysiological mechanism. Proteotoxic stress in desmin and phospholamban cardiomyopathy is present in a relatively high percentage of cardiomyocytes in the myocardium as compared to most other groups of genetic cardiomyopathies. p62 was diffusely present in the myocardium, not related to fibrosis. These findings might suggest that the accumulation of proteotoxic aggregates in the sarcolemma is the first hit in the pathophysiology of desmin and phospholamban cardiomyopathy, making the cell less stable and vulnerable for a second hit such as local mechanical stress

We also observed a high percentage of p62‐positive aggregates in phospholamban cardiomyopathy, which is caused by the *PLN* p. Arg14del mutation. This Dutch founder mutation is the most prevalent cardiomyopathy‐related genetic variant in the Netherlands, being present in 12% of patients clinically diagnosed with ACM and 15% of patients with DCM.^15^ The mutation is also incidentally found in patient populations in the USA, Canada, Germany, Greece and China.^16^, ^17^, ^18^ Phospholamban regulates the sarcoplasmic reticulum Ca2+‐ATPase pump (SERCA2a) thereby determining the rate of relaxation and contraction of the cardiac myocyte. The p. Arg14del mutation is believed to result in super‐inhibition of SERCA2a inducing decreased calcium transport from the cytosol back into the sarcoplasmic reticulum during diastole. We have previously demonstrated that phospholamban‐positive cardiomyocyte aggregates are specific for *PLN* p. Arg14del cardiomyopathy and can be used in diagnostic pathology.^5^, ^19^, ^20^ In the present study, we show that proteotoxic stress in phospholamban cardiomyopathy is present in a relatively large percentage of cardiomyocytes in the myocardium as compared to most other genetic cardiomyopathies. Strikingly we did not find a correlation between p62‐positive aggregates and fibrosis in phospholamban cardiomyopathy. p62 was more diffusely present in the myocardium, whereas we have previously shown that fibrosis is predominantly observed in the RV wall and the LV posterolateral wall,^8^, ^9^ areas with high strain during exercise. The combination of these findings suggests that the accumulation of proteotoxic aggregates that replace parts of the contractile elements in the sarcolemma is the first hit in the pathophysiology of phospholamban cardiomyopathy, leading to instable cells which are vulnerable for a second hit such as local mechanical stress (Figure 4). Another disease mechanism that has been suggested to play a role in phospholamban cardiomyopathy is activation of calmodulin‐dependent kinase II and calcineurin A due to disturbed calcium handling, which may lead to maladaptive remodelling of the macromolecular protein complex that forms the intercalated disc.^21^

Also in desmosmal mutation‐positive arrhythmogenic cardiomyopathies, p62‐positive aggregates were observed. This is in line with observations in a recent desmoplakin (*DSP*) mouse model where hearts not only showed focal fat infiltrations in the RV (consistent with arrhythmogenic cardiomyopathy), but also cytoplasmic aggregates consisting of intercalated disc proteins such as desmoplakin, plakoglobin and connexin 43. These aggregates coincided with disruption of the intercalated discs, intermediate filaments and microtubules.^22^ Desmosomal proteins are connected by protein‐protein complexes to intermediate filaments such as desmin.^10^ It was therefore suggested that these aggregates were a direct result of a ‘final common pathway’ disturbance of the interplay between desmosomal proteins and desmin, with the final phenotype consistent with a desminopathy.^22^ Interestingly, we observed a lower percentage of p62‐positive aggregates in the RV as compared to the septum and LV in hearts/patients with desmosomal gene mutations. A possible explanation might be that in arrhythmogenic cardiomyopathy RV cardiomyocytes show increased decay with replacement of myocardium by fibrosis and adipocytes. As the aggregates coincide with the disruption of intercalated discs, cardiomyocytes containing aggregates are probably prone to a faster decay than cardiomyocytes with intact intercalated discs and without aggregates in this type of cardiomyopathy.

The hearts of the other patient groups revealed the lowest percentages of p62‐positive cardiomyocytes that were comparable to the p62 levels of patients without known pathogenic mutations. Sarcomeric mutations are usually missense and nonsense variants which cause a dysfunctional protein that can still be integrated in the sarcomere of the cardiomyocyte. This might explain why in patients with sarcomeric mutations accumulation of mutated proteins and p62‐positivity appears to be limited. In the sarcomeric group, 3 patients had a variant of unknown significance (2 *MYH7* and 1 *TNNI3*); therefore, the cardiomyopathy in these patients might have been affected by other variables that we have currently not yet identified. Also, in lamin A/C hearts we observed relatively low percentages of p62‐positive cardiomyocytes which is in concordance with a mouse model study where the increase of p62‐positive cells was only twofold compared to controls.^23^ Overall, in cardiomyopathy hearts an increase of p62‐positive aggregates was observed as compared to controls, also in hearts without known pathogenic mutation. Therefore, a slightly diminished autophagic degradation due to increased proteotoxic stress might be a more general phenomenon of diseased cardiomyocytes.

A possible limitation of this study is the relatively low number of hearts per mutation group. We advise that in the near future cardiovascular pathologists around the world should work in collaboration with cardiologists and clinical geneticists to create databases and biobanks of cardiomyopathy patients that include clinical variables, genetic variants and detailed examination of cardiac tissue. In order to accomplish this, it is important that pathologists work according to standardized protocols when examining hearts and initiatives to create such a protocol (eg under the auspices of the Society for Cardiovascular Pathology and the Association of European Cardiovascular Pathology) are urgently needed. Furthermore, we used explanted hearts, and therefore, our results only represent end‐stage disease.

In conclusion, accumulation of p62‐positive protein aggregates is homogeneously distributed in the myocardium independently of fibrosis distribution and strongly associated with desmin and phospholamban cardiomyopathy. Proteotoxic protein accumulation probably is a diffuse process in the myocardium where a second hit, such as local strain during exercise, might determine whether this leads to regional myocyte decay and fibrosis.

## CONFLICT OF INTEREST

The authors confirm that there are no conflicts of interest.

## AUTHOR CONTRIBUTION

**Joy van der Klooster:** Conceptualization (equal); Data curation (lead); Formal analysis (lead); Methodology (equal); Project administration (equal); Resources (equal); Software (equal); Validation (equal); Visualization (equal); Writing‐original draft (lead). **Shahrzad Sepehrkhouy:** Data curation (equal); Methodology (equal); Software (equal); Writing‐review & editing (equal). **Dennis Dooijes:** Data curation (equal); Resources (equal); Writing‐review & editing (equal). **Wouter te Rijdt:** Data curation (equal); Methodology (equal); Resources (equal); Writing‐review & editing (equal). **Frederique Schuiringa:** Data curation (equal); Investigation (equal); Writing‐review & editing (equal). **Jolanthe Lingeman:** Data curation (equal); Investigation (equal); Writing‐review & editing (equal). **Peter van Tintelen:** Resources (equal); Supervision (equal); Validation (equal); Writing‐review & editing (equal). **Magdalena Harakalova:** Funding acquisition (equal); Investigation (equal); Software (equal); Writing‐review & editing (equal). **Roel Goldschmeding:** Supervision (equal); Writing‐review & editing (equal). **Albert Suurmeijer:** Conceptualization (equal); Methodology (equal); Supervision (equal); Writing‐review & editing (equal). **Folkert Asselbergs:** Funding acquisition (lead); Resources (equal); Supervision (equal); Writing‐review & editing (equal). **Aryan Vink:** Conceptualization (lead); Data curation (equal); Formal analysis (equal); Investigation (equal); Methodology (equal); Project administration (equal); Resources (equal); Software (equal); Supervision (equal); Validation (equal); Visualization (equal); Writing‐review & editing (equal).

## Supporting information

Table S1

## Data Availability

Data are available upon request because of privacy/ethical restrictions.
