# Supplementary material for: P62‐positive aggregates are homogenously distributed in the myocardium and associated with the type of mutation in genetic cardiomyopathy
Source: J Cell Mol Med. 2021 Feb 18;25(6):3160–6. doi: 10.1111/jcmm.16388 (PMC7957157; doi:10.1111/jcmm.16388)
Supplement: Supplementary file 1 — Table S1 [file JCMM-25-3160-s001.pdf]

Supplementary table to:

P62 positive aggregates are homogenously distributed in the myocardium and associated with the type of mutation in genetic cardiomyopathy.

Van der Klooster ZJ, Sepehrkhouy S, Dooijes D, Te Rijdt WP, Schuiringa FSAM, Lingeman J, van Tintelen JP, Harakalova M, Goldschmeding R, Suurmeijer AJH, Asselbergs FW and Vink A.

Journal of Cellular and Molecular Medicine

Supplemental table 1. Genetic variants (mutations) of the genetic cardiomyopathy patients

	Gene	Variant	Mutation protein	Type of mutation	ACMG classification	Explant / Autopsy
1	*DES*	NM_001927.3(DES):c.1310del	p.Gly437Valfs*10	Frameshift	Likely pathogenic (PVS1, PM2)	E
2	*DES*	NM_001927.3(DES):c.1024A>G	p.Asn342Asp	Missense/splice variant	Pathogenic (PS2, PS3, PS4, PM2, PP1, PP2)	A
3	CRYAB	NM_001289807.1(CRYAB):c.527A>G	p.*176Trpext*19	Read-through	Pathogenic (PS3, PM2, PM4, PP1, PP3, PP4)	E
4	*PLN*	NM_002667.3(PLN):c.40_42del	p.Arg14del	In-frame deletion	Pathogenic (PS3, PS4, PM2, PM4, PP1, PP4)	E
5	*PLN*	NM_002667.3(PLN):c.40_42del	p.Arg14del	In-frame deletion	Pathogenic (PS3, PS4, PM2, PM4, PP1, PP4)	E
6	*PLN*	NM_002667.3(PLN):c.40_42del	p.Arg14del	In-frame deletion	Pathogenic (PS3, PS4, PM2, PM4, PP1, PP4)	E
7	*PLN*	NM_002667.3(PLN):c.40_42del	p.Arg14del	In-frame deletion	Pathogenic (PS3, PS4, PM2, PM4, PP1, PP4)	E
8	*PLN*	NM_002667.3(PLN):c.40_42del	p.Arg14del	In-frame deletion	Pathogenic (PS3, PS4, PM2, PM4, PP1, PP4)	E
9	*PLN*	NM_002667.3(PLN):c.40_42del	p.Arg14del	In-frame deletion	Pathogenic (PS3, PS4, PM2, PM4, PP1, PP4)	E
10	*PLN*	NM_002667.3(PLN):c.40_42del	p.Arg14del	In-frame deletion	Pathogenic (PS3, PS4, PM2, PM4, PP1, PP4)	A
11	*PLN*	NM_002667.3(PLN):c.40_42del	p.Arg14del	In-frame deletion	Pathogenic (PS3, PS4, PM2, PM4, PP1, PP4)	A
12	*PLN*	NM_002667.3(PLN):c.40_42del	p.Arg14del	In-frame deletion	Pathogenic (PS3, PS4, PM2, PM4, PP1, PP4)	A
13	PKP2	NM_004572.3(PKP2):c.2386T>C	p.Cys796Arg	Missense	Pathogenic (PS3, PS4, PM2, PP1, PP2, PP3)	E
14	*PKP2*	NM_004572.3(PKP2):c.235C>T	p.Arg79*	Nonsense	Pathogenic (PVS1, PS3, PS4, PM2, PP1)	E
15	*PKP2*	NM_004572.3(PKP2):c.2544G>A	p.Trp848*	Nonsense	Pathogenic (PVS1, PS3, PS4, PM2, PP1)	E
16	*PKP2*	NM_004572.3(PKP2):c.397C>T	p.Gln133*	Nonsense	Pathogenic (PVS1, PS3, PS4, PM2, PP1)	E
17	*DSP*	NM_004415.2(DSP):c.4078dup	p.(Ile1360Asnfs*2)	Frameshift	Likely Pathogenic (PVS1, PM2)	A
18	*DSP*	NM_004415.2(DSP):c.1705A>T	p.Lys569*	Nonsense	Likely Pathogenic (PVS1, PM2)	E
19	*LMNA*	NM_170707.2(LMNA):c.308A>G	p.Gln103Arg	Missense	Pathogenic (PS4, PM2, PP1, PP2, PP3, PP4)	A
20	*LMNA*	NM_170707.2(LMNA):c.308A>G	p.Gln103Arg	Missense	Pathogenic (PS4, PM2, PP1, PP2, PP3, PP4)	E
21	*LMNA*	NM_170707.2(LMNA):c.949G>A	p.Glu317Lys	Missense	Pathogenic (PS4, PM2, PP1, PP2, PP3, PP4)	E
22	*LMNA*	NM_170707.2(LMNA):c.308A>G	p.Gln103Arg	Missense	Pathogenic (PS4, PM2, PP1, PP2, PP3, PP4)	E
23	*LMNA*	NM_170707.2(LMNA):c.992G>A	p.Arg331Gln	Missense	Pathogenic (PS4, PM2, PM5, PP1, PP2, PP3, PP4)	E
24	*LMNA*	NM_170707.2(LMNA):c.1130G>T	p.Arg377Leu	Missense	Pathogenic (PS4, PM2, PM5, PP1, PP2, PP3, PP4)	E
25	*LMNA*	NM_001282625.1 (LMNA):c.-318-?_119+?del	p.Met1_Arg119del	Deletion	Pathogenic (PVS1, PM2, PM4)	E
26	*MYBPC3*	NM_000256.3(MYBPC3):c.2827C>T	p.Arg943*	Nonsense	Pathogenic (PVS1, PS3, PS4, PM2, PP1)	E
27	*MYBPC3*	NM_000256.3(MYBPC3):c.2864_2865del	p.(Pro955Argfs*95)	Frameshift	Pathogenic (PVS1, PS3, PS4, PM2, PP1)	E
28	*MYH7*	NM_000257.2(MYH7):c.704C>G	p.Thr235Ser	Missense	VUS (PM2, PP2, PP3)	E
29	*MYH7*	NM_000257.2(MYH7):c.2945T>C	p.Met982Thr	Missense	Benign (BA1, BS4, BP5)	E
	MYH7	NM_000257.2(MYH7):c.4172A>G	p.Lys1391Thr	Missense	VUS (PM2, PP2, PP3)	E
30	*TNNI3*	NM_000363.4(TNNI3):c.292C>T	p.Arg98*	Nonsense	Pathogenic (PS3, PS4, PM2, PP1)	E
31	*TNNI3*	NM_000363.4(TNNI3):c.539A>G	Asp180Gly	Missense	VUS (PM2, PP2, PP3)	E
32	*TNNT2*	NM_000364.4(TNNT2):c.650_652del	p.Lys217del	In frame deletion	Pathogenic (PS1, PS3, PS4, PM2, PP2)	E
